# Effect of Bendopnea on Achievement Medical Treatment Target Doses in Heart Failure

**DOI:** 10.34172/aim.2023.06

**Published:** 2023-01-01

**Authors:** Gülsüm Meral Yılmaz Öztekin, Ahmet Genç, Anıl Şahin, Göksel Çağırcı, Şakir Arslan

**Affiliations:** ^1^Department of Cardiology, Antalya Training and Research Hospital, University of Health Sciences, Antalya, Turkey; ^2^Department of Cardiology, Sivas Cumhuriyet University, Sivas, Turkey

**Keywords:** Bendopnea, Heart failure, Medical treatment

## Abstract

**Background:**

The newly described bendopnea in heart failure (HF) is associated with increased cardiac filling pressures. The aim of the study was to show the effect of bendopnea follow-up on reaching optimal medical treatment doses in HF.

**Methods:**

A total of 413 patients were screened, and we included 203 patients with HF who were previously evaluated for bendopnea. Demographic data, presence or absence of bendopnea, medical history, laboratory findings, and medical treatments were evaluated. Optimal medical therapy target doses at baseline and 3rd month were compared in groups with and without bendopnea.

**Results:**

On admission, 64 patients (31.5%) had bendopnea. The rate of patients with bendopnea decreased in the 3rd month (n=42, 20.7%). The proportion of patients who used at least 50% of the recommended medical treatment dose on admission and in the 3rd month was compared; angiotensin-converting enzyme inhibitor /angiotensin receptor blockers use increased from 40.6% to 71.9% in those with bendopnea (*P*=0.013), from 56.1% to 81.3% in those without bendopnea (*P*<0.001) and beta-blockers use increased from 28.2% to 60.9% in those with bendopnea (*P*=0.042), from 31.6% to 69.8% in those without bendopnea (*P*<0.001). However, aldosterone antagonists use decreased from 70.3% to 67.2% in those with bendopnea (*P*=0.961), from 68.4 % to 64.1% in those without bendopnea (*P*=0.334). Bendopnea was independently effective in achieving ACE-I/ARB target doses (OR: 0.359, CI 95%: 0.151–0.854, *P*=0.020).

**Conclusion:**

Bendopnea follow-up in HF patients can provide a significant improvement in reaching the recommended treatment target doses.

## Introduction

 Heart failure (HF) affects more than 23 million patients worldwide and causes increased morbidity and mortality.^1^ Despite significant advances in the treatment of HF, the mortality rate still remains high.^2,3^

 The traditional typical symptoms of HF are shortness of breath, orthopnoea, paroxysmal nocturnal dyspnea, and reduced exercise tolerance. It is recommended that the symptoms and signs of HF should be carefully evaluated at each outpatient visit and treatment changes should be made according to the signs of congestion.^4^ Bendopnea in HF was first defined in 2014 as shortness of breath on bending forward in less than 30 seconds while wearing or tying shoes.^5^ Bendopnea is the result of an increase in cardiac filling pressures and is associated with a worse cardiac index and higher right heart pressures.^5^

 The frequency of bendopnea in the general population without HF has been reported as 6.7%.^6^ While the frequency of bendopnea is 18% in outpatient cases with systolic HF, its frequency increases to 48.8% in decompensated HF.^7,8^ Bendopnea has been shown to be associated with short-term mortality, especially in advanced New York Heart Association (NYHA) functional class (III and IV).^8^

 HF guidelines recommend the use of angiotensin-converting enzyme inhibitors or angiotensin receptor blockers (ACE-I/ARBs), beta-blockers, aldosterone antagonists, ivabradine, and, more recently, angiotensin receptor–neprilysin inhibitor (ARNI) at maximally tolerated target doses to reduce mortality and/ or rehospitalizations due to HF. These treatment approaches provide better outcomes and survival in HF.^4,9^ Physicians’ adherence to treatment guidelines was also shown to be a strong predictor of fewer hospitalizations in actual practice.^10^ However, only 30% of HF patients are treated with the targeted maximum tolerated dose of these drugs.^11^

 As far as we know, there is no article in the literature on the use of bendopnea in the follow-up of HF patients and its relationship with medical treatments. We aimed to show the effect of bendopnea on reaching optimal medical treatment doses at 3 months in HF.

## Materials and Methods

 Patients aged > 18 years and with a left ventricular ejection fraction (LVEF) ≤ 50% who were followed up regularly were included in the study. Since 2019, bendopnea is routinely evaluated during patient examination in the HF outpatient clinic of our hospital. The presence or absence of bendopnea and onset time are recorded. Bendopnea is evaluated by measuring the time it takes to feel short of breath while sitting in a chair as if tying one’s shoes.^5^ If there is a symptom before 30 seconds, the presence of bendopnea is considered and the onset time is recorded in the patient’s file. Between February 2019 and March 2020, 413 patients who were evaluated for bendopnea were screened, and 203 patients with at least 3 months of follow-up were included in the study. Patients with severe lung disease were excluded from the study. Sixty-four patients with bendopnea and 139 patients without bendopnea were compared. Demographic data, physical examination, HF symptoms (dyspnea, orthopnea, edema), bendopnea onset time, NYHA functional classification, electrocardiogram, echocardiography, medical history, medical treatments, and laboratory findings were evaluated retrospectively from the clinical database of the HF outpatient clinic. In the 3rd month, the presence of bendopnea, medical treatments, and doses were re-evaluated based on the medical records. All laboratory parameters were measured with standard methods. The SPSS software version 22.0 was used for statistical analysis. Kolmogorov-Smirnov test and Q-Q plot were used for normality. Homogeneity of variance was tested by Levene’s method. Normally distributed results were expressed as means with standard deviations and Student’s *t* test was used. Non-normally distributed results were expressed as medians and interquartile range (IQR). Number and percentage were used for categorical variables. Continuous variables that were not normally distributed were analyzed by Mann-Whitney U test to compare the clinical features of the groups with and without bendopnea. Chi-square test was used for categorical variables. The chi-square test was used when no more than 20% of the expected numbers were less than 5 and all individual expected counts were 1 or greater. Mean difference and odds ratio estimates with 95% confidence intervals (CIs) were used for the association between bendopnea and medical therapy target doses (with Fisher’s exact test, two-way ANOVA, and Greenhouse–Geisser correction). Multivariate logistic regression analysis was performed to adjust for potential confounders. A *P* value < 0.05 was considered statistically significant.

## Results

 The median age of the participants, of whom 69% (n: 140) were male and 31% (n: 63) were female, was 62 years [IQR 55–71]. The median LVEF of patients was 30% [IQR 25–35] and 27% (n: 55) were NYHA functional classes III and IV. Bendopnea was present in 64 of the patients (31.5%) and it was absent in 139 patients (68.5%). The onset time of bendopnea was 18 seconds [IQR 13–25]. Dyspnea was the most common symptom (74.9%, n: 152), followed by orthopnea in 25.1% (n: 51) and edema in 15.8% (n: 32).

 The basic characteristics of the patients according to bendopnea status are shown in Table 1. Bendopnea was found more frequently in women than men (*P* < 0.001). In addition, dyspnea, orthopnea, and edema complaints were observed more frequently in the bendopnea group (*P*< 0.001). Resting heart rate and atrial fibrillation (AF) rates were slightly higher in those with bendopnea (respectively, *P* = 0.051, *P*= 0.057). Most patients with bendopnea were NYHA class III and IV (n:33, 51.6%). When the laboratory findings were compared, albumin and hemoglobin levels were lower in the bendopnea group (respectively, *P* = 0.003, *P* < 0.001), while N-terminal pro-brain natriuretic peptide (NT-proBNP) was higher (*P* = 0.028). C-reactive protein was slightly higher (*P*= 0.048). Hypertension, diabetes, ischemic etiology and echocardiographic findings were similar between the groups. While the NT-proBNP values of the patients on admission were 1844 [671–3837] ng/L, they decreased to 1200 [453–3097] ng/L in the 3rd month (*P* = 0.528).

**Table 1:** Baseline Characteristics of Heart Failure Patients According to Bendopnea Status

**Characteristics**	**With Bendopnea (n=64)**	**Without Bendopnea (n=139)**	***P*** ** Value**
Age (y)	65 (58–72)	62 (54–71)	0.073
Male, n (%)	32 (50)	108 (77.7)	< 0.001
BMI (kg/m^2^)	29 (25–33)	26 (24–30)	0.013
HF duration (mon)	18 (6–84)	12 (3–42)	0.093
Dyspnea, n (%)	62 (96.9)	90 (64.7)	< 0.001
Orthopnea, n (%)	30 (46.9)	21 (15.1)	< 0.001
Edema, n (%)	21 (32.8)	11 (7.9)	< 0.001
Ischemic etiology of heart failure, n (%)	34 (53.1)	79 (56.8)	0.621
Medical history			
Diabetes, n (%)	26 (40.6)	51 (36.7)	0.591
Hypertension, n (%)	37 (57.8)	70 (50.4)	0.323
Atrial fibrillation, n (%)	17 (26.6)	19 (13.7)	0.057
Systolic blood pressure (mm Hg)	110 (110–120)	110 (100–130)	0.684
Diastolic blood pressure (mm Hg)	60 (60–70)	60 (60–70)	0.797
Heart rate (b.p.m)	82 (68–92)	74 (65–86)	0.051
NYHA I, n (%)	2 (3.1)	46 (33.1)	< 0.001
NYHA II, n (%)	29 (45.3)	71 (51.1)	
NYHA III and IV, *n* (%)	33 (51.6)	22 (15.8)	
Echocardiography			
LV EDD (mm)	55 (50–61)	58 (53–63)	0.063
LV ESD (mm)	45 (41–53)	48 (43–54)	0.129
PAB (mmHg)	44 (31–55)	37 (30–48)	0.110
LA diameter (mm)	45 (41–51)	45 (40–49)	0.359
LVEF, %	30 (25–33)	30 (20–35)	0.663
Laboratory			
eGFR (mL/min/1,73 m^2^)	66 (48–76)	69 (53–82)	0.125
Creatinine (mg/dL)	1.07 (0.92–1.27)	1.09 (0.97–1.31)	0.390
Sodium (mmol/L)	139 (137–141)	139 (137–141)	0.581
Albumin (g/dL)	4.4 (4–4.6)	4.6 (4.3–4.8)	0.003
Haemoglobin (g/dL, mean ± SD)	12.6 ± 1.69	13.6 ± 1.78	< 0.001
High sensitive troponin T (ng/L)	19 (14–34)	19 (12–32)	0.568
C-reactive protein (mg/dL)	5.1 (2.1–12.5)	3.6 (1.6–8.2)	0.048
NT-proBNP (ng/L)	2481 (824–5324)	1535 (551–3340)	0.028

BMI, body mass index; b.p.m., beats per minute; NYHA, New York Heart Association; LV EDD, left ventricular end-diastolic diameter; LV ESD, left ventricular end-systolic diameter; PAB, pulmonary artery pressure; LA, left atrium; LVEF, left ventricular ejection fraction; eGFR, estimated glomerular filtration rate; NT-proBNP, N-terminal pro-brain natriuretic peptide. Normally distributed data are presented as mean + SD. Non-normally distributed data are presented as median (interquartile range). eGFR was calculated using Chronic Kidney Disease Epidemiology Collaboration (CKD-EPI) equation.

 At the end of the 3rd month, the rate of bendopnea decreased significantly compared to the baseline, and bendopnea was observed in 42 patients (31.5% vs 20.7%, *P* < 0.001). When the medical treatments recommended by the guidelines were evaluated, 82.3% (n: 167) of all patients were taking ACE-I/ARBs, 90.1% (n: 183) were taking beta-blockers and 74.4% (n: 151) were taking aldosterone antagonists on admission. The rate of medical treatment recommended by the guidelines increased at 3 months compared to baseline. 96.6% (n: 196) of the patients were taking ACE-I/ARBs, 98.5% (n: 200) beta-blockers and 87.2% (n: 177) aldosterone antagonists at the 3rd month (respectively, *P* < 0.001, *P* = 0.564, *P* = 0.002). The proportion of all patients who achieved at least 50% of the target doses in the 3rd month, ACE-I/ARBs (58.4% vs. 78.3%, *P*< 0.001) and beta-blockers (30.5% vs. 67%, *P* < 0.001) increased significantly. However, a slight reduction in aldosterone antagonists was also seen (68.9% vs. 65.1%, *P* = 0.044). The proportion of those receiving less than 50% of the target dose increased from 3.1% to 25% in those with bendopnea and from 5.8% to 20.9% in those without bendopnea compared to baseline (respectively, *P*= 0.962, *P* = 0.331). Diuretic use increased from 61.6% to 63.1% (*P* < 0.001).

 The patients who received at least 50% of the treatment target doses recommended by the guidelines followed up with bendopnea were evaluated on admission and 3rd months (Table 2). The proportions of patients receiving at least 50% of the target dose in the bendopnea group increased significantly for ACE-I/ARBs and beta-blockers, but not for aldosterone antagonists (respectively, from 40.6% to 71.9% *P* = 0.013, from 28.2% to 60.9% *P* = 0.042, from 70.3% to 67.2%, *P* = 0.961). ACE-I/ARBs use increased from 56.1% to 81.3% (*P*< 0.001) and beta-blockers use increased from 31.6% to 69.8% (*P*< 0.001). However, aldosterone antagonist use decreased from 68.4 % to 64.1% in those without bendopnea (*P*= 0.334). Loop diuretic use was higher in those with bendopnea at both time points (Table 2). The proportion of patients who received at least 50% of the target dose for ACE-I/ARB increased significantly at 3 months (OR F(1,1):225, 95% CI: 137.9–217.9, *P* = 0.042) (Table 2). Multivariate logistic regression analysis was performed to adjust for confounders, including bendopnea, age, HF duration, NYHA class, hypertension, education, hospitalization for HF, chronic kidney disease. Bendopnea was independently effective in achieving ACE-I/ARB target doses (OR: 0.359, CI 95%: 0.151–0.854, *P* = 0.020) (Table 3).

**Table 2:** Medical Treatments and NT-proBNP Levels of Heart Failure Patients with and without Bendopnea on Admission and at 3rd Month

**Medications**	**On Admission**		***P *** **Value**	**At 3rd Month**		***P *** **Value**	**OR (95% CI)**	***P *** **Value**
	**With Bendopnea**	**Without Bendopnea**		**With Bendopnea**	**Without Bendopnea**			
ACE-I/ARB, n (%)	46 (71.9)	121 (87.1)	0.009	60 (93.8)	136(97.8)	0.138	0.861 (0.549–1.358)	0.563
< 50% target doses	20 (31.3)	42 (30.2)	0.041	14 (21.9)	23 (16.5)	0.287	F(1,1):225 (137.9–217.9)	0.042
≥ %50 target doses	26 (40.6)	78 (56,1)		46 (71.9)	113 (81.3)			
Beta-blockers, n (%)	56 (87.5)	127 (91.4)	0.390	62 (96.8)	138 (99.3)	0.187	1.449 (0.899–2.308)	0.125
< 50% target doses	37 (57.8)	82 (59)	0.626	23 (35.9)	41 (29.5)	0.420	F(1,1):0.526 (17.44–55.56)	0.600
≥ %50 target doses	18 (28.2)	44 (31.6)		39 (60.9)	97 (69.8)			
Loop diuretics, n (%)	48 (75)	77 (55.4)	0.008	49 (76.6)	79 (56.8)	0.007	1.263 (0.737-2.107)	0.423
Aldosterone antagonist, n (%)	47 (73.4)	104 (74.8)	0.834	59 (92.2)	118 (84.9)	0.148	0.903(0.572–1.455)	0.722
< 50% target doses	2 (3.1)	8 (5.8)	0.423	16 (25)	29 (20.9)	0.424	F(1,1):25 (111.9–65.94)	0.125
≥ %50 target doses	45 (70.3)	95 (68.4)		43 (67.2)	89 (64.1)			
ARNI, n (%)	4 (6,3)	2 (1.4)	0.060	4 (6,3)	2 (1.4)	0.060		
NT-proBNP (ng/L)	2481 (823–5324)	1535 (550–3340)	0.028	1632 (504–3555)	1165 (420–2909)	0.117		

ACE-I, angiotensin-converting enzyme inhibitor; ARB, angiotensin receptor blocker; ARNI, angiotensin receptor/neprilysin inhibitor; NT-proBNP, N-terminal pro-brain natriuretic peptide; OR, odds ratio; CI, confidence interval.

**Table 3:** Multivariable Logistic Regression for Potential Confounders Related to Reaching the Medical Therapy Target Doses

**Variables**	**ACE-I/ARB**			**Beta-Blocker**			**Aldosterone Antagonist**		
	**β**	**OR (95% CI)**	***P*** ** Value**	**β**	**OR (95% CI)**	***P*** ** Value**	**β**	**OR (95% CI)**	***P*** ** Value**
Bendopnea	-1.025	0.359 (0.151–0.854)	0.020	0.077	1.080 (0.450–2.590)	0.863	-0.176	0.839 (0.330–2.132)	0.712
Age	-0.003	0.997 (0.967–1.028)	0.850	-0.009	0.991 (0.960–1.023)	0.575	-0.025	0.975(0.941–1.011)	0.169
HF duration	-0.004	0.996 (0.988–1.003)	0.263	0.002	1.002 (0.995–1.010)	0.513	0.000	1.000(0.992–1.011)	0.943
NYHA class	0.496	1.642 (0.654–4.124)	0291	-0.266	0.766 (0.293–2.007)	0.588	-0.381	0.683 (0.253–1.848)	0.453
Hypertension	0.581	1.788 (0.839–3.811)	0.132	0.728	2.071 (0.932–4.603)	0.074	-0.778	0.459 (0.201–1.050)	0.065
Education	1.261	3.528(0.471–26.427)	0.220	1.485	4.416 (0.574–33.977)	0.552	0.210	1.234 (0.142–10.752)	0.849
Hospitalization for HF	0.364	1.439 (0.864–2.397)	0.162	0.027	1.028 (0.608–1.738)	0.919	0.142	1.152 (0.676–1.964)	0.602
Chronic kidney disease	-0.645	0.525 (0.145–1.902)	0.326	-0.658	0.518 (0.131–2.044)	0.347	-1.351	0.259 (0.072–0.929)	0.038

ACE-I, angiotensin-converting enzyme inhibitor; ARB, angiotensin receptor blocker; OR, odds ratio; CI, confidence interval; HF, heart failure; NYHA, New York Heart Association.

**Figure 1:**
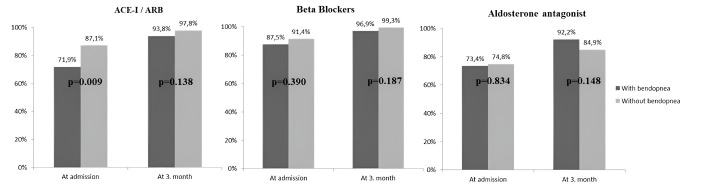
Medical Treatments of Heart Failure Patients with and without Bendopnea on Admission and at 3rd Month. (ACE-I/ARB, angiotensin-converting enzyme inhibitor/angiotensin receptor blocker)

**Figure 2:**
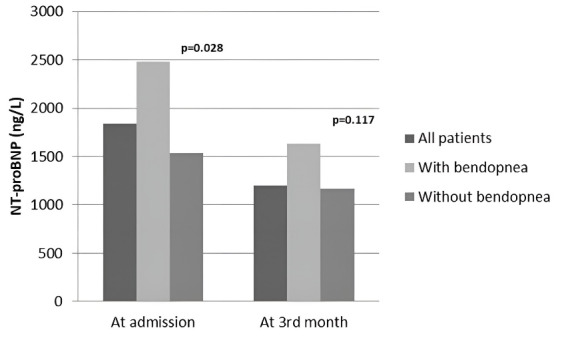
NT-proBNP levels of Patients with and without Bendopnea at Admission and 3rd Month. (NT-proBNP, N-terminal pro-brain natriuretic peptide)

 The medical treatments and NT-proBNP levels of the patients on admission and the 3rd month according to bendopnea status are shown in Figure 1. The use of ACE-I/ARBs was significantly lower in those with bendopnea on admission in both groups. However, the rate of using beta blockers and aldosterone antagonists was similar. In the 3rd month, the rate of ACE-I/ARBs, beta-blockers, and aldosterone antagonists increased similarly in both the bendopnea and non-bendopnea groups (Figure 1). NT-proBNP levels were higher in the bendopnea group on admission (*P*= 0.028); and decreased in both groups in the 3rd month. However, it was similar in the two groups (*P* = 0.117) (Table 2 and Figure 2).

## Discussion

 In this study, we reported that the evaluation of bendopnea in the follow-up of HF can provide a significant improvement in reaching the recommended medical treatment doses. The bendopnea rate decreased from 31.5% to 20.7% at the end of 3 months of follow-up. At 3 months, the rate of reaching at least 50% of the target doses of ACE-I/ARBs and beta-blockers, which are known to reduce mortality, increased significantly. The proportion of patients who received at least 50% of the target dose for ACE-I/ARB increased significantly at 3 months. Bendopnea was independently effective in achieving ACE-I/ARB target doses in multivariate analyses. Evaluation of bendopnea in the follow-up of HF patients can provide a stronger idea about the congestion status and filling pressures. Therefore, assessment of bendopnea may compel clinicians to achieve optimal medical therapy doses of HF treatments.

 Dyspnea that occurs within 30 seconds of bending forward while putting on or tying shoes has been recently reported in 2014 as a new symptom called bendopnea in HF. The already high ventricular filling pressure in HF patients is further increased by this maneuver.^5^ The study by Thibodeau et al included mostly elderly patients (mean age 81.8 years), with a frequency of approximately 48.8% and a mean time of onset of bendopnea of 13.4 seconds ( ± 6.9).^5^ In our study, the median age was 62 years, the frequency of bendopnea was 31.5%, and the onset time of bendopnea was 18 seconds [IQR 13-25]. With age, the frequency of bendopnea may increase and the onset of shortness of breath may be earlier. However, it is necessary to carry out studies specific to age ranges to support this notion. Because that study included patients presenting with decompensated HF, the frequency was higher compared to our study, which included more stable outpatients. In another study with stable ambulatory HF, bendopnea was reported with a lower frequency (18%).^7^

 Dyspnea was the most common symptom in our study population (74.9%), and this rate was higher in those with bendopnea (96.9%). Orthopnea was present in 46.9% and edema in 32.8% of patients with bendopnea. Although this rate was lower than the study on patients with decompensated HF (59% and 70.5%, respectively), it was significantly higher than patients without bendopnea. The patients with bendopnea described dyspnea as the most frequent symptom, at 99.2%.^8^ While dyspnea is reported as the most common symptom of hospitalization, paroxysmal nocturnal dyspnea is associated with increased left ventricle filling pressures.^12,13^ Edema is one of the most common symptoms of decompensated HF, but several noncardiac causes can produce it.^12^ In a meta-analysis, there was an association between bendopnea, dyspnea, orthopnea, and paroxysmal nocturnal dyspnea.^14^ Dyspnea, orthopnea and edema complaints were more common in patients with bendopnea in our study, too. Therefore, we believe that the evaluation of the presence of bendopnea can provide more objective data about the increase in filling pressures.

 Similarly, in previous studies, hypertension and diabetes were not associated with bendopnea.^5^ The presence of AF and heart rate was slightly higher in patients with bendopnea in our study. Confirming this, bendopnea shows more symptomatic patients. The interaction between body mass index (BMI) and bendopnea is not clear. In our study and the first bendopnea study with higher BMI values, BMI and bendopnea were found to be associated.^5^ However, in another study with similar BMI values to our study, no relationship was found with bendopnea.^9^

 More than half of HF patients with bendopnea were in NYHA class III and IV in our study. Similarly, bendopnea has been previously associated with functional class NYHA III and IV, indicating it is a symptom of advanced HF.^5,7^ However, physical examination is essential in the diagnosis and treatment of HF and can provide as much insight into cardiac filling pressures as brain natriuretic peptides.^4^ NT-proBNP levels were higher in the bendopnea group on admission. At 3 months, this difference disappeared and there was a similar decrease in NT-proBNP values in both groups. Therefore, we believe that bendopnea evaluated together with NYHA classes and NT-proBNP can yield a better idea about filling pressures.

 In a previous study, the rates of ACE-I/ARBs, beta-blockers, and mineralocorticoid receptor antagonists used in the outpatient population with HF with reduced LVEF were 90.5%, 87.8%, and 42.7%, respectively, but the number of patients using drugs at the target dose was below 50%,^15^ whereas the same rates were 82.3%, 90.1%, and 74.4%, respectively, in our study. However, in our follow-up, the rate of patients who received 50% of the target dose increased significantly in ACE-I/ARBs and beta-blockers compared to admission (respectively, 58.4% vs. 78.3%, *P* < 0.001, 30.5% vs. 67%, *P* < 0.001). There was a significant reduction for the aldosterone antagonists (68.9% vs. 65.1%, *P* = 0.044). The proportion of those who received less than 50% of the target dose of aldosterone antagonists increased from baseline in both those with and without bendopnea. This may be due to higher doses of ACE-I/ARB in follow-up and the reduction or discontinuation of aldosterone antagonists due to the resulting hyperkalemia.

 As far as we know, the doses of HF treatments and the proportion of patients reaching the target dose in patient groups with and without bendopnea have not been reported before. In the 3rd month, the rate of use of ACE-I/ARBs, beta-blockers, and aldosterone antagonists increased in all patients with and without bendopnea. The proportion of those who received ACE-I/ARBs and at least 50% of the target dose was significantly lower in the bendopnea group both on admission and in the 3rd month. More loop diuretics were used in patients with bendopnea at both time points, as it was associated with higher filling pressures. However, in the first study on bendopnea, medical treatment rates were similar between the groups with and without bendopnea, including diuretics.^5^ In a study on outpatient cases with systolic HF, the rates of non-diuretic treatments were found to be similar between the groups with and without bendopnea.^7^ Also, the rate of aldosterone antagonist use in our study was 93.3%, while it was approximately 50% in the other two studies.^5,7^ In our study, the rate of those who received at least 50% of the target dose of ACE-I/ARBs and beta-blockers at 3 months increased in HF patients with and without bendopnea.

 Our study had several limitations. The number of patients was limited since the study was retrospectively planned in a single center and patients previously evaluated for bendopnea were included. A prospective study with a longer follow-up period can be performed with a larger number of patients. In our study, treatments were directed based on NYHA classes or NT-proBNP levels or bendopnea status during follow-up and the rates of reaching the target dose were not compared. Since our study was a retrospective observational study, we could not provide these data. Prospective studies can be planned to compare HF follow-up methods and determine which one is more applicable. Since the LVEF of the patients in our study was ≤ 40%, there is insufficient data for HF with mid-range and preserved LVEF. It is difficult to determine which drug has a greater bendopnea-reducing effect, as it is recommended to start with these drugs that reduce mortality in HF treatment guidelines. Such an evaluation could not be made because the drugs were not started separately.

 In conclusion,the use of bendopnea in the follow-up of HF patients can provide an objective understanding of the severity of symptoms. We also believe that it may be challenging for us to reach optimal medical treatment doses. Evaluation of bendopnea in addition to symptoms, NYHA classes, and NT-proBNP levels during follow-up can provide objective data in patient follow-up and accelerate the achievement of treatment goals.
